# Editorial: Pediatric Neurometabolic Disorders

**DOI:** 10.3389/fneur.2021.737398

**Published:** 2021-09-07

**Authors:** Brahim Tabarki, Juan Dario Ortigoza-Escobar, Wang-Tso Lee, Majid AlFadhel

**Affiliations:** ^1^Division of Neurology, Department of Pediatrics, Prince Sultan Military Medical City, Riyadh, Saudi Arabia; ^2^Movement Disorders Unit, Institut de Recerca Sant Joan de Déu, CIBERER-ISCIII and European Reference Network for Rare Neurological Diseases (ERN-RND), Barcelona, Spain; ^3^Department of Pediatrics, National Taiwan University Hospital, Taipei, Taiwan; ^4^King Abdullah International Research Center (KAIMRC), Medical Genomics Research Department, King Saud Bin Abdulaziz University for Health Sciences, King Abdulaziz Medical City, Ministry of National Guard Health Affairs, Riyadh, Saudi Arabia; ^5^Division of Genetics, Department of Pediatrics, King Abdullah Specialized Children's Hospital, King Abdulaziz Medical City, Ministry of National Guard Health Affairs, Riyadh, Saudi Arabia

**Keywords:** inherited neurometabolic disorders, movement disorders, leukodystrophy, stroke, mitochondrial disease

Inherited neurometabolic disorders represent a growing group of inborn errors of metabolism and many are potentially treatable. These inborn errors of metabolism are distinctly heterogeneous, both clinically and genetically. Advances in genetics have revolutionized the way we understand, diagnose and manage these inherited neurometabolic disorders. To date, more than 1,450 disorders have been included in the International Classification of Inherited Metabolic Disorders (ICIMD). Care for pediatric patients with neurometabolic disorders is, therefore, a rapidly expanding subspecialty in neurology. Early detection and early intervention in these disorders are invaluable in achieving normal or near-normal neurodevelopmental milestones for many patients.

(1) Anderson et al. provide a research article on novel insights into the monitoring of Ornithine transcarbamylase deficiency, focusing on the contribution of physiological processes and neurocognitive function in this population.
(2) Hu et al. retrospectively reviewed the clinical presentation, pathological features, genetic characteristics, and follow up of a cohort of mitochondrial myopathy in children from China, and preliminarily analyzed the risk factors and treatments correlated with the prognosis.
(3) Ortigoza-Escobar provides an overview of inborn metabolic errors that present movement disorders, suggests red flags and diagnostics clues for suspecting inborn errors of metabolism, and proposes minimum biochemical studies as stated in each movement disorder and the differential diagnoses according to the neuroradiological findings, providing evidence on symptomatic or disease specific-treatment through a six-step algorithm.
(4) Tabarki et al. review pediatric-onset metabolic disorders with Mendelian and mitochondrial inheritance and predominant spinal cord involvement. They provide an overview of these conditions, including background information and examples that require rapid identification, focusing on treatable conditions that would be catastrophic if they are not recognized.
(5) Tabarki et al. review reported literature on the inherited metabolic causes of stroke in children, focusing on mechanisms, types, and management.
(6) Alfadhel et al. retrospectively reviewed the spectrum of leukodystrophy in Saudi Arabia based on a multicentre study. A detailed description of the epidemiological, clinical, radiological, and genetic data of leukodystrophies is described.
(7) Hu et al. reported two Chinese patients with mitochondrial encephalopathy due to *FOXRED1* mutations. They also did an extensive literature search on the same disorder.
(8) Almannai et al. present an overview of metabolic seizures based on various criteria such as treatability, age of onset, seizure type, and pathogenetic background.

These manuscripts represent an exciting and insightful snapshot of current knowledge of inherited neurometabolic disorders in children. State-of-the-art, existing challenges and emerging future topics are highlighted in this special issue.

## Author Contributions

All authors listed have made a substantial, direct and intellectual contribution to the work, and approved it for publication.

## Conflict of Interest

The authors declare that the research was conducted in the absence of any commercial or financial relationships that could be construed as a potential conflict of interest.

## Publisher's Note

All claims expressed in this article are solely those of the authors and do not necessarily represent those of their affiliated organizations, or those of the publisher, the editors and the reviewers. Any product that may be evaluated in this article, or claim that may be made by its manufacturer, is not guaranteed or endorsed by the publisher.

